# Non-canonical retinoid signaling in neural development, regeneration and synaptic function

**DOI:** 10.3389/fnmol.2024.1371135

**Published:** 2024-03-07

**Authors:** Alicia Piazza, Robert Carlone, Gaynor E. Spencer

**Affiliations:** Department of Biological Sciences, Brock University, St. Catharines, ON, Canada

**Keywords:** retinoic acid, non-genomic, neurite outgrowth, synaptic plasticity, kinases, growth cones

## Abstract

Canonical retinoid signaling via nuclear receptors and gene regulation is critical for the initiation of developmental processes such as cellular differentiation, patterning and neurite outgrowth, but also mediates nerve regeneration and synaptic functions in adult nervous systems. In addition to canonical transcriptional regulation, retinoids also exert rapid effects, and there are now multiple lines of evidence supporting non-canonical retinoid actions outside of the nucleus, including in dendrites and axons. Together, canonical and non-canonical retinoid signaling provide the precise temporal and spatial control necessary to achieve the fine cellular coordination required for proper nervous system function. Here, we examine and discuss the evidence supporting non-canonical actions of retinoids in neural development and regeneration as well as synaptic function, including a review of the proposed molecular mechanisms involved.

## 1 Introduction to canonical retinoid signaling

Retinoic acid (RA) is synthesized via a two-step enzymatic reaction (Duester, 2008) from Vitamin A (retinol), obtained prenatally through the placenta or postnatally in the diet (Clagett-Dame and DeLuca, 2002). The receptors that bind RA are members of the nuclear hormone receptor family and thus regulate gene transcription. They include retinoic acid receptors (RARs) (Giguère et al., 1987; Petkovich et al., 1987) and retinoid X receptors (RXRs) (Hamada et al., 1989; Mangelsdorf et al., 1990) and three different subtypes for each RAR and RXR (α, β, γ) have been cloned from human and murine genomes (Petkovich and Chambon, 2022). One additional RAR subtype, RAR δ, appears to be unique to urodele amphibians (Ragsdale et al., 1989). Alternative splicing and use of different gene promoters result in the generation of multiple isoforms for each retinoid receptor subtype (e.g., RARα1-2; RARβ1-4; RARγ1-2; Kastner et al., 1990; Leroy et al., 1991a,b; Zelent et al., 1991; Ragsdale et al., 1993). Two different isoforms for each of the three RXR subtypes have also been identified (Petkovich and Chambon, 2022).

RAR binds both all*-trans* RA (atRA, the main biologically active retinoid) and its isomer, 9-*cis* RA (Heyman et al., 1992; Levin et al., 1992). 9-*cis* RA was initially considered the endogenous ligand for RXR (Allenby et al., 1993), but this is now thought to be 9-*cis*-13-14-dihydroretinoic acid, at least in mice (Rühl et al., 2015). During canonical retinoid signaling, RA binding induces conformational changes in the receptors to promote RXR/RAR heterodimerization. This heterodimer binds to a DNA sequence called the retinoic acid-response element (RARE) located in the enhancer/promoter region of RA-responsive genes, and together with coactivator recruitment, facilitates retinoid-mediated transcriptional control (Chen and Evans, 1995; Zamir et al., 1996; le Maire et al., 2010). The specific isoform of each receptor can ultimately dictate transcriptional regulation.

A ligand is not required for retinoid receptor-DNA binding but is critical for determining whether an RXR/RAR complex activates or silences target gene expression (Mangelsdorf and Evans, 1995). Unliganded RAR primarily interacts with a corepressor complex to repress transcription of specific gene promoters (Chen and Evans, 1995; Kurokawa et al., 1995; Zamir et al., 1996); such receptor-mediated gene silencing is important in controlling gene expression during various developmental processes (Weston et al., 2003; Kumar and Duester, 2014). RAR-bound genes can be actively transcribed before RA exposure (Mahony et al., 2011), also suggesting constitutive actions of RAR on gene expression.

## 2 Introduction to non-canonical retinoid signaling

In this review, we focus on non-canonical retinoid signaling, which includes retinoid receptor-independent and receptor-dependent (non-genomic) signaling mechanisms. Specifically, we consider non-canonical signaling to include any *direct* effects of RA on cell signaling cascades *independently* of retinoid receptors. These effects might include signaling as a result of RA binding to and activating cellular retinoic acid binding proteins (CRABPs). Non-canonical retinoid signaling can also include processes that involve RA binding to retinoid receptors (RAR or RXR), often located outside the nucleus, resulting in non-genomic signaling (no direct transcriptional regulation by heterodimerized receptors; Taylor and Heyland, 2022). Such non-canonical actions of RA and/or the receptors can occur outside of the nucleus, including locally in axons and dendrites.

We will discuss evidence for various non-canonical effects of RA in the development, regeneration and plasticity of neurons in the nervous system. It should be noted that many non-canonical effects have been examined with exogenous application of RA and exactly which factors might ultimately control such effects still requires investigation, though some advances in this area have been made (Aoto et al., 2008; Wang et al., 2011). It is also not yet clear how canonical and non-canonical actions of RA might interact, though instances where physiological processes might involve (or require) both are discussed in more detail below.

It is important to distinguish between non-canonical and non-genomic signaling as there might be instances where retinoid signaling is considered non-canonical in nature but lacks evidence to rule out involvement of transcriptional regulation. For example, many effects of RA are considered non-genomic based only on the speed of RA's actions, yet the time required to complete transcription is highly dependent on gene length (Kirkconnell et al., 2017). As such, it is important that the requirement for transcription is tested directly, rather than inferred from the timescale of the biological effect (Gelfman et al., 2012; Fuchs et al., 2014; Veloso et al., 2014).

## 3 Non-canonical retinoid signaling and kinase interactions

An early report of non-canonical retinoid signaling involved the identification of a putative RA binding site on the intracellular regulatory enzyme, protein kinase C (PKC) (Radominska-Pandya et al., 2000). This discovery led to the concept of receptor-independent retinoid signaling, in which retinoids might exert their effects independently of their canonical receptors, RAR and RXR, or at the very least, independently of nuclear-localized RAR/RXR.

Early biochemical evidence for direct atRA-PKCα interactions showed that radiolabeled [^3^H] atRA was displaced from purified PKC by atRA, 9-*cis* RA and other retinoid metabolites (Radominska-Pandya et al., 2000; Ochoa et al., 2003). Crystallization experiments then indicated that atRA could bind to both the calcium binding site and lysine rich residue of PKCα, the latter being the main phospholipid binding site (Ochoa et al., 2003). It was determined that atRA competed with small molecules that bind to this site, such as the phospholipid PS (phospholipid phosphatidyl-L-serine) (Radominska-Pandya et al., 2000). AtRA (1 μM) was most effective when not competing with PS, and higher doses of atRA were required if PS was already bound. These results indicated that atRA binds with high affinity to PKC, but the presence of competing small molecules (such as PS) modulates atRA's ability to alter PKC activity (Radominska-Pandya et al., 2000). Because PS is involved in inducing the translocation of cytosolic PKCα to the membrane where it becomes activated, it was proposed that the competitive binding of atRA (to the same sites as PS) might prevent PKCα translocation, leading to an overall reduction in cellular PKC activity (Radominska-Pandya et al., 2000). However, RA has also been shown to activate various isoforms of PKC (Sparatore et al., 2000; Miloso et al., 2004; Chan et al., 2012), though such activation may be context-dependent, or indirect (as evidence suggests it often involves other kinases or CRABPs; Wei, 2016).

## 4 Development: a brief overview of the role of retinoids

Vitamin A-deficient avian and mammalian embryos have presented with impairments in axonal elongation, aberrant trajectory of developing cranial nerves and lack of neurite outgrowth from the spinal cord to the periphery (Maden et al., 1996; White et al., 1998, 2000a,b; Clagett-Dame et al., 2006). The importance of Vitamin A and retinoid signaling in developmental processes was confirmed with retinoid receptor mutants (which produced similar effects as Vitamin A deficiency). Indeed, the advancement of molecular genetic approaches allowed the induction of stage-specific deficiencies, providing insight into the importance of RA at specific developmental timepoints (Teletin et al., 2023). There is unique RAR expression during different stages of development (Huang et al., 2008; Linville et al., 2009) and retinoid signaling is under tight spatiotemporal control via regulation of the bioavailability of RA for receptor binding (Duester, 2008). Enzymes involved in RA production (retinaldehyde dehydrogenases; RALDHs) and its subsequent breakdown (cytochrome P450 enzymes) display unique, non-overlapping tissue-specific expression during embryogenesis. This creates RA gradients now known to be important for early patterning (Horton and Maden, 1995; Mic et al., 2002).

Most developmental effects of retinoids were considered a result of canonical regulation of gene transcription, though non-canonical signaling processes are emerging. There is now evidence that non-canonical effects of RA (e.g., via kinases and CRABP) can affect cell fate decisions and neuronal differentiation. However, the extent of non-canonical interactions of RA with other well-known developmental signals, such as Wnt (Wingless/Integrated), Shh (Sonic hedgehog), or Hippo, is not yet clear. Recent evidence however, suggests that RA non-genomically interacts with Notch signaling (Larange et al., 2023), which is known to co-ordinate differentiation into distinct cell fates.

### 4.1 Non-canonical retinoid interactions with kinases and implications for cellular proliferation and differentiation

#### 4.1.1 CRABP1: a messenger for non-canonical retinoid-kinase interactions

Whereas, CRABP2 delivers RA to nuclear retinoid receptors (for gene transcription), CRABP1 directly binds RA to mediate either receptor-independent signaling (Gupta et al., 2008; Persaud et al., 2013; Lin et al., 2017, 2021), or signaling requiring interactions between CRABP1 and cytoplasmically-localized RAR (Cañón et al., 2004; Persaud et al., 2013). However, CRABP1 also shows a nuclear localization in mouse embryos and various cell lines (Gaub et al., 1998), and though there is no evidence for direct interactions with nuclear RARs/RXRs, it may possibly regulate nuclear retinoid receptor activity (Noy, 2000; Wei, 2016).

CRABP1 can mediate interactions between RA and various kinases; for example, the effects of RA on calcium-calmodulin-dependent protein kinase II (CaMKII) involve CRABP1 (Tsai et al., 2015; Lin et al., 2022). CRABP1 also mediates interactions between atRA and mitogen-activated protein kinase (MAPK/ERK) and does so independently of RAR (Gupta et al., 2008; Persaud et al., 2013; Lin et al., 2017). Other studies suggest a requirement for RAR in MAPK/ERK activation, based on evidence that (a). RAR-selective ligands also induce kinase activity (Cañón et al., 2004; Khatib et al., 2019a) (b). RAR complexes with upstream kinase regulators (Piskunov and Rochette-Egly, 2012; Tsai et al., 2015), and (c). there are concomitant changes in RAR expression and kinase activity (Tsai et al., 2015). The involvement of retinoid receptors was also shown in RA-induced non-genomic modulation of PI3K (phosphoinositide 3 kinase) (Masiá et al., 2007; Tsai et al., 2015), and non-canonical modulation of Rho-kinases (Dey et al., 2007).

**Figure 1:**
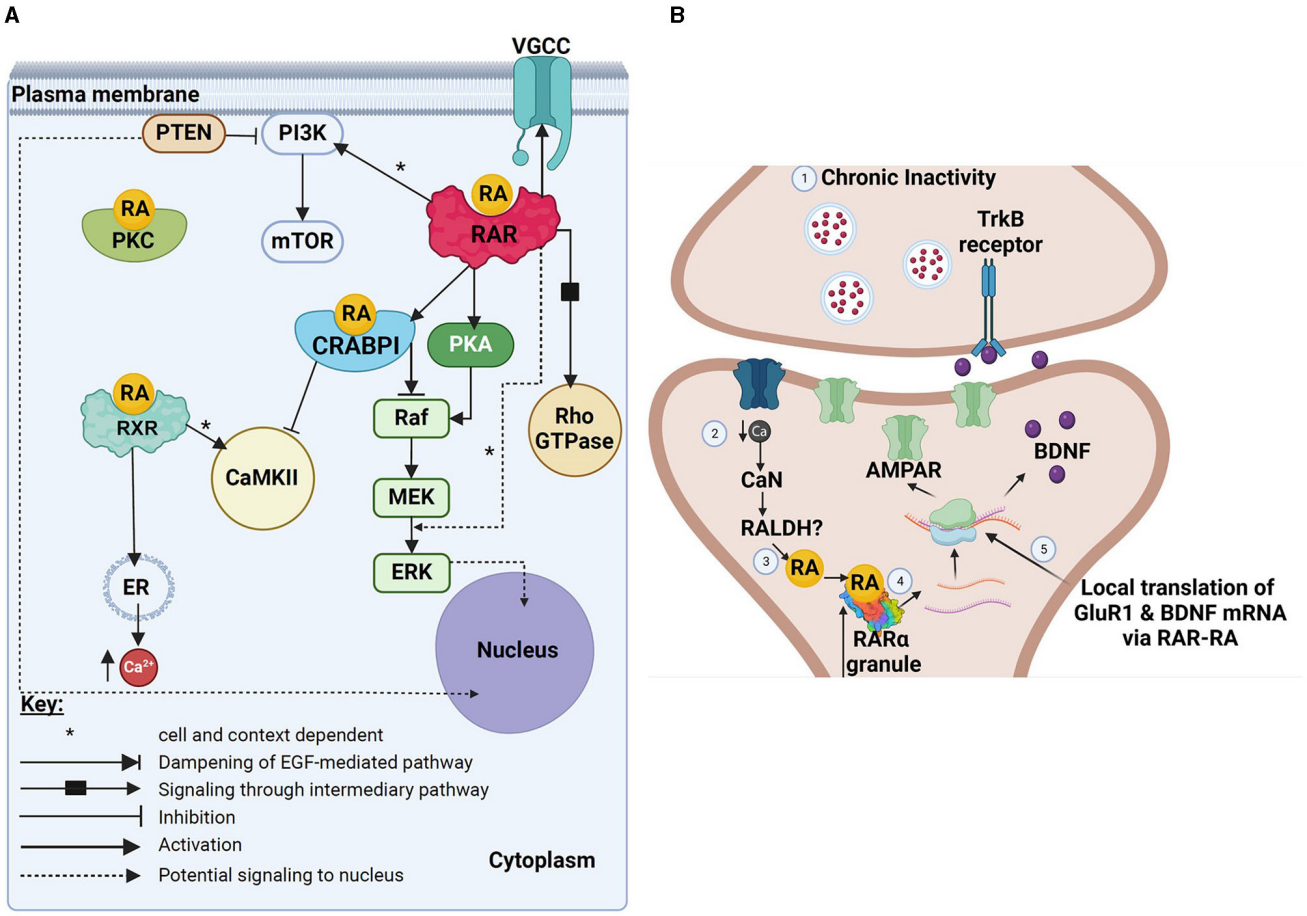
Schematic summary of various non-canonical actions of RA. **(A)** Image showing various non-canonical actions potentially mediated by either RA alone (direct actions), RA binding to CRABP1, or RA binding to extranuclear RAR or RXR receptors. Black boxes represent possible intermediary pathways (either not yet known or not yet described fully, or eliminated here for reasons of simplicity), whereas dotted lines represent potential downstream effects of various effector molecules in the nucleus (and might represent eventual downstream transcriptional effects). **(B)** A schematic summary of the known effects of RA and RARα in the translational control of GluR1 and BDNF in mediating homeostatic synaptic plasticity. Pre-synaptic (top) and post-synaptic (bottom) sites are shown. This figure has been adapted from Thapliyal et al. (2022). Created with BioRender.com. **(A)** VGCC, voltage-gated calcium channels; PI3K, phosphoinositide 3 kinase; PTEN, neuronal phosphatase tensin homolog; RA, retinoic acid; PKC, protein kinase C; mTOR, mechanistic target of rapamycin; RAR, retinoic acid receptor; RXR, retinoid X receptor; CRABPI, cellular retinoic acid binding protein 1; PKA, protein kinase A; Rho-GTPase, Ras homologous GTPase; Raf, rapidly accelerated fibrosarcoma kinase; MEK, mitogen-activated protein kinase; ERK, extracellular regulated kinase; ER, endoplasmic reticulum; Ca^2+^, calcium. **(B)** TrKB, Tropomyosin receptor kinase B; Ca, calcium; CaN, calcineurin; RALDH, retinal dehydrogenase; RA, retinoic acid; RARα, retinoic acid receptor alpha; AMPAR, α-amino-3-hydroxy-5-methyl-4-isoxazolepropionic acid receptor; BDNF, brain-derived neurotrophic factor; GluR1, glutamate-receptor subunit-1.

It is worth noting that protein kinases directly activated by RA or CRABP1 (non-canonical signaling) might eventually translocate to the nucleus and phosphorylate different target proteins (including nuclear retinoid receptors, histone proteins, splicing regulatory and RNA binding proteins), ultimately participating in downstream transcriptional regulation of RA-target genes (Bruck et al., 2009; Laserna et al., 2009; Meseguer et al., 2013; Piskunov et al., 2014). Together, this suggests that rapid, non-canonical actions of RA might play a role in the integration of cytoplasmic signals in the nucleus (see Figure 1A). Kinases also initiate phosphorylation cascades that lead to downstream control of mRNA translation (Meseguer et al., 2013; Tsai et al., 2015) or alternative splicing (Laserna et al., 2009), both of which can play a role in post-transcriptional changes in gene expression during RA-induced neuronal differentiation.

#### 4.1.2 Non-canonical kinase interactions in stem cell fate decisions

Studies on the regulation of stem cell cycles, from proliferation to differentiation, have provided insights into how non-canonical RA signaling is associated with kinase activity. In early-stage differentiation of cultured human trophoblast cells, atRA (10 μM) rapidly (within 5 min) controls local translation of RARβ and RXRα mRNA. This involves receptor-independent retinoid activation of PI3K/Akt3/mammalian target of rapamycin (mTOR), which prevents the assembly of a cap-dependent mRNA translation repression complex (eIF4E-4EBP1), allowing the translation of RARβ and RXRα (Tsai et al., 2015). This then leads to translation-related reductions in mRNA levels (Khuperkar et al., 2020), a process unaffected by transcriptional inhibitors (Tsai et al., 2015), thus confirming a non-genomic mechanism.

The newly synthesized RARβ and RXRα proteins were then shown to complex with Gβ and Gαq_11_ subunits, respectively (Tsai et al., 2015). RARβ-Gβ non-canonically regulates the c-Src/c-Raf/p-MEK/p-Erk1/2/CREB1 pathway, whereas RXRα-Gαq_11_ regulates calcium-calmodulin-dependent protein kinase II (CaMKII) (Tsai et al., 2015). RA-mediated signaling via retinoid receptor-G protein complexes was examined between 1 and 24 h, but in the absence of transcriptional/translational inhibitors. Thus, despite the demonstration of non-genomic control of receptor translation, it is currently unclear whether the non-canonical activation of the downstream kinase cascades is also non-genomic in nature.

Retinoid receptor coupling with G-proteins has also been reported elsewhere; RARs form complexes with G-protein subunits in cultured mammalian cancer cells (Piskunov and Rochette-Egly, 2012; Rochette-Egly, 2015) and human platelets (Moraes et al., 2007). Invertebrate neuronal RARs might also interact with G proteins to rapidly and non-canonically mediate voltage-dependent inhibition of calcium channels (de Hoog et al., 2019).

#### 4.1.3 Rapid ERK kinase activity during stem cell fate commitment is RAR-independent

Embryonic stem cells treated with low concentrations of exogenous RA (100 nM) show a bi-phasic activation of ERK1/2. Initial RA-mediated activation occurred within 30 min and was independent of RAR, suggesting an initial non-canonical activation of ERK1/2 by atRA. CRABP-ERK mediates the phosphorylation of the nuclear protein p27 (cyclin-independent kinase interacting protein kinase inhibitor, involved in cell cycling), thus promoting retention of nuclear p27 and arresting cell growth to prepare these stem cells for differentiation. The second, slower activation phase of ERK 1/2 (at 8–12 h), was, however, mediated by RAR (Persaud et al., 2013).

It was later shown that CRABP1-dependent atRA signaling modulated neural stem cell differentiation into neurospheres and was a critical modulator of neural stem cell pool expansion in the mouse hippocampus (Lin et al., 2017). More recently, Raf kinases were identified as targets of atRA-CRABP1 in embryonic stem cells (Park et al., 2019). AtRA-CRABP1 competes with and dampens epidermal growth factor (EGF) activation of the Ras/Raf/Mek/ERK pathway, thus slowing the cell cycle progression to favor cell differentiation. This contrasts with EGF-dominant activation, which results in cell proliferation (Knight et al., 2019; Park et al., 2019).

Non-canonical retinoid activity during neuronal differentiation is not limited to ERK1/2 phosphorylation, but also includes rapid activation of the PI3K signaling pathway, at least in SH-SY5Y cells (López-Carballo et al., 2002; Masiá et al., 2007). Cells treated with 1 μM atRA or 9-*cis* RA, showed rapid and specific phosphorylation of downstream Akt kinase (Ser_473_), independently of translation or transcription. Triple RAR knockout (and restoration) did, however, determine a requirement for RARs in RA-induced PI3K signaling (Masiá et al., 2007). PI3K activity also plays an important role in RA-dependent mRNA translational control, highlighting a post-transcriptional role for RA-kinase interactions in cellular differentiation (Laserna et al., 2009).

#### 4.1.4 Post-transcriptional regulatory mechanisms of RA signaling during development

RA signaling is a known epigenetic regulator during differentiation, acting through various mechanisms, including regulation of chromatin dynamics during transcription (Urvalek and Gudas, 2014; Wu et al., 2024). RA is also involved in non-canonical post-transcriptional regulation, such as mRNA processing, including mRNA splicing (Laserna et al., 2009) and production of non-coding RNAs (ncRNAs) (García-Padilla et al., 2021). Below, we discuss the significance of ncRNAs as regulatory molecules in RA-induced differentiation and brain development.

As mentioned previously, activation of PI3K by atRA (or 9-*cis* RA) is required for differentiation of neuroblastoma cells (López-Carballo et al., 2002). Altered phosphoprotein populations were also identified in neuroblastoma cells acutely treated with atRA (1 μM, 30 min). These included mostly splicing regulatory proteins (Laserna et al., 2009; Meseguer et al., 2011) and it was shown that RA influenced splice site selection and increased the number of spliced isoforms. This non-genomic RA signaling was dependent on both RAR and PI3K signaling, but was independent of transcription (Laserna et al., 2009). RA has also been shown to upregulate a ncRNA (microRNA) that, in turn, causes a transition in pre-mRNA alternate splicing patterns from non-neuronal to neuronal, promoting neuronal differentiation (Makeyev et al., 2007). However, whether this relationship between RA and microRNA is non-canonical in nature is not yet known. The role of retinoid signaling in mRNA processing is reviewed more extensively by Meseguer et al. (2013).

MicroRNAs (miRNA) are small ncRNAs that once mature, post-transcriptionally regulate gene expression by either repressing mRNA translation or degrading target mRNAs (Bartel, 2004; Chekulaeva and Filipowicz, 2009). MiRNAs can target hundreds of different mRNAs, allowing for unique temporal and spatial control of mRNA translation. The vertebrate brain has a high expression of miRNAs, highlighting the importance of post-transcriptional regulation in nervous system function (Miska et al., 2004; Sempere et al., 2004; Lau and Hudson, 2010). MiRNA expression profiles have now identified specific miRNAs important for various RA-induced processes (reviewed in Gholikhani-Darbroud, 2020; Wang et al., 2020), including neuronal differentiation (Le et al., 2009; Annibali et al., 2012; Hu et al., 2017; Gao et al., 2019; You et al., 2020), axonal outgrowth (Su et al., 2020), and CNS organization (Qin et al., 2016; Liu et al., 2020).

RA likely promotes miRNA biogenesis through canonical retinoid receptor-dependent transcriptional activation (Hu et al., 2019) as RAREs have been found upstream of miRNA promoters (albeit in some non-neuronal cells) (Lee et al., 2017; Hu et al., 2019). However, RA can also induce miRNA gene transcription via other transcription factors (Fazi et al., 2005; Huang et al., 2010; Nurrahmah et al., 2021), the activation of which might involve non-canonical RA signaling. RA might also alter the activity of existing pre-miRNAs through *de novo* miRNA biogenesis or inhibition of miRNA maturation. MiRNA maturation can be accelerated by neural activity, which together with calcium influx, can activate miRNA synthesizing machinery (Dicer) (Lugli et al., 2005). Interestingly, neuronal activity and calcium influx are known to affect RA levels (Aoto et al., 2008), and in turn, be directly affected by RA (Vesprini et al., 2015; de Hoog et al., 2018, 2019; de Hoog and Spencer, 2022).

## 5 Neurite outgrowth and regeneration: a brief overview of the role of retinoids

The role of RA in the induction or enhancement of neurite outgrowth was initially described by examining cultured rat spinal cord neurons, axolotl and newt spinal cord explants and mouse dorsal root ganglion cultures (Wuarin et al., 1990; Hunter et al., 1991; Corcoran et al., 2000; Dmetrichuk et al., 2005). A number of studies examining the effects of retinoids on neurite outgrowth have utilized cultured neurons. Some of these include embryonic neurons (Maden et al., 1998; Rand et al., 2017) and might thus represent developmental programs, whereas others use cultured adult neurons (resulting from axotomy) and thus more closely represent an injury model (Dmetrichuk et al., 2006). Despite this, neurite outgrowth from cultured neurons (even adult neurons) might not truly represent regeneration, which is generally considered the re-growth of nerves *in situ* toward their targets, leading to functional recovery. We do, however, include culture studies in our discussions of neurite outgrowth and regeneration below.

Adult CNS neurons in vertebrates are generally not capable of functional regeneration, aside from certain species including (but not limited to) zebrafish (Blum and Begemann, 2012) and some urodele amphibians, including newts and axolotls (Hunter et al., 1991; Tanaka and Ferretti, 2009). This capacity for regeneration in some adult vertebrates is proposed to be partly due to the upregulation of retinoid signaling components in response to injury (Carter et al., 2011; Nguyen et al., 2017), which fails to occur in the CNS of most other adult vertebrates (Corcoran et al., 2002). For example, RA signaling is critical for the initiation of the blastema (regeneration-competent progenitor cell pool that acquires the identity of the lost appendage), and the upregulation and/or activation of retinoid signaling machinery is required for regeneration of newt tail and limb and axolotl spinal cord (Blum and Begemann, 2012; Monaghan and Maden, 2012; McCusker et al., 2015). For details of canonical retinoid signaling in regeneration, refer to the review by Maden (2020).

### 5.1 Non-canonical role of retinoids in neurite outgrowth and regeneration

The physiological effects of retinoids are classically characterized based on their genomic activity and it is well-established that retinoid-mediated gene transcription is critical for neurite outgrowth (Clagett-Dame et al., 2006). However, non-genomic effects of retinoids may also be contributing to such physiological functions (previously characterized as genomic), including the induction of neurite outgrowth. For example, Khatib et al. (2019a) utilized a gene expression assay together with an ERK1/2 phosphorylation assay, to study mechanisms involved in the neurite-inducing capacity of synthetic and natural retinoids. Only ligands capable of activating both gene transcription and ERK1/2 phosphorylation enhanced neurite outgrowth from SH-SY5Y cells (even at very low concentrations of 10 nM). Synthetic retinoids that activated only gene expression, or only ERK1/2 phosphorylation, promoted the formation of shorter neurites.

Though ERK1/2 activity was tested 30 min after application of atRA, gene activity was tested at 24 h. The minimal concentration of atRA required to induce gene activation was found to be 10,000-fold lower (0.0001 nM) than the concentration required to induce ERK1/2 phosphorylation (1 nM). The authors thus proposed that the non-genomic and genomic actions of retinoids were likely regulated through independent pathways. This has also been suggested for other nuclear hormone receptors; Smith et al. (2008) proposed that a unique set of receptors and signaling pathways, distinct from those regulating transcription, likely mediate the non-genomic effect of progesterone.

The potential involvement of unidentified retinoid receptor isoforms or retinoid binding proteins in the non-genomic induction of neurite outgrowth should also be considered. Though shown in T-cells (not neurons) Larange et al. (2023) identified an extranuclear isoform of RARα; this cytoplasmic RARα lacks the DNA-binding domain and nuclear translocation sequence, though retains the ligand-binding domain. The authors rendered the nuclear RARα non-functional (by overexpression of a dominant-negative form) and demonstrated the novel cytoplasmic isoform's independent contribution to retinoid signaling. Direct binding of RA to the ligand binding domain of this novel cytoplasmic receptor isoform still, however, requires confirmation.

#### 5.1.1 Retinoid receptor localization in neurites and growth cones and their role in axon guidance

**Figure 2:**
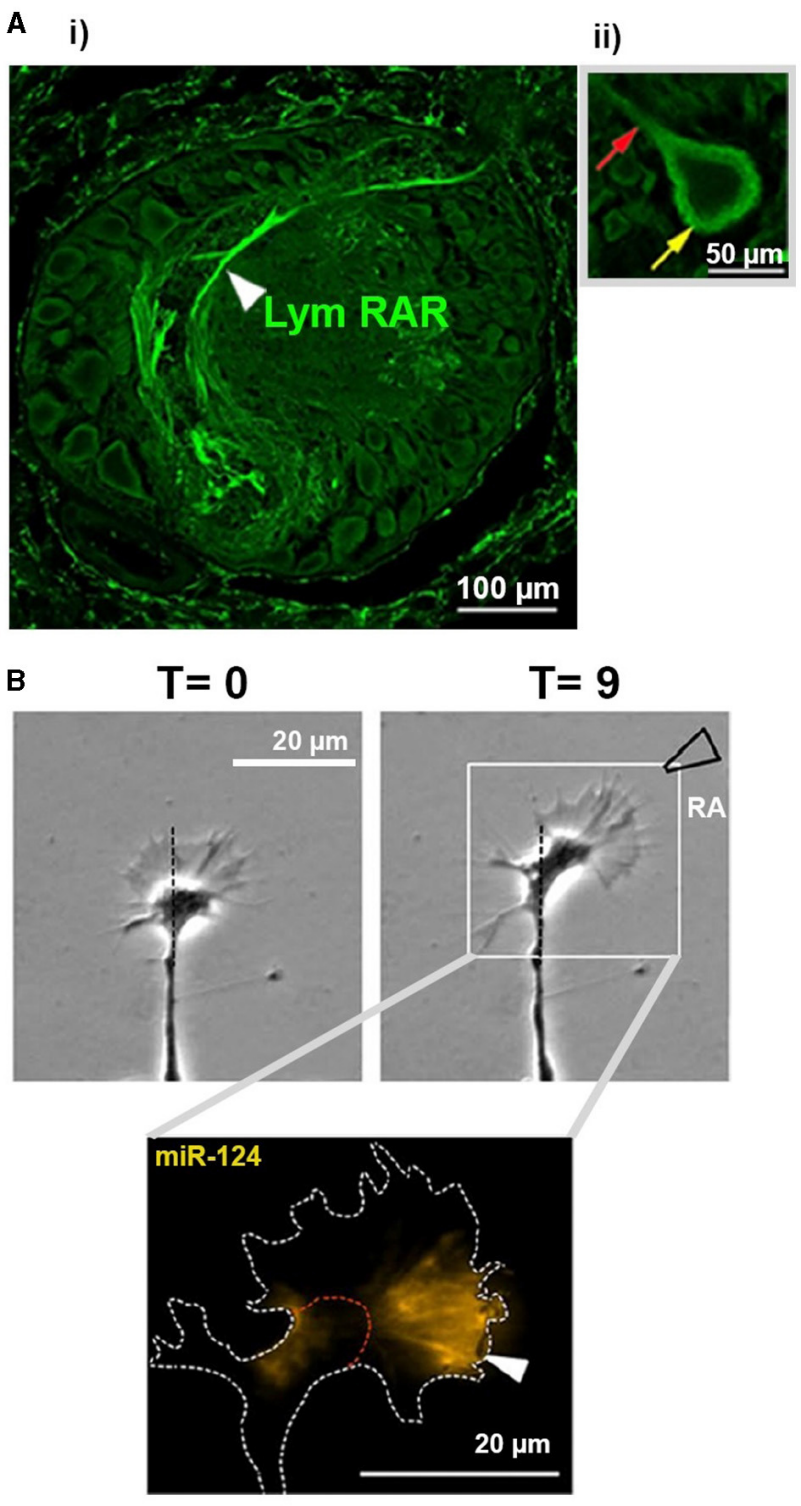
Images from invertebrate CNS and cultured cells examining non-canonical effects of RA. **(A)** (i) Image showing RAR immunostaining within one ganglion of the *Lymnaea* CNS. Cell bodies are shown around the edge of the ganglion, from which primary neurites emerge and enter the center of the ganglion (neuropil). Note the extensive staining for RAR in the neuropil regions. (ii) There is no staining for RAR observed within the nuclei (central domain) of a cell body. Yellow arrow indicates extensive staining in the cytoplasmic region of the cell, as well as some staining detectable in the primary neurite emerging from the cell body (red arrow). Images taken from Carter et al. (2015) © 2014 Wiley Periodicals, Inc. **(B)** Time-lapse phase-contrast images (top) of a growth cone from a cultured *Lymnaea* motorneuron exhibiting rapid turning (chemoattraction) toward a local source of atRA (approx. region of pipette indicated). *In situ* hybridization (bottom) indicating the localization of miR-124 (yellow) in the growth cone following its response to RA. Images taken and modified from Walker et al. (2022); Copyright © 2020, Springer Science Business Media, LLC, part of Springer Nature. Any changes to the original published images were performed using Adobe Photoshop (2020), used only to remove original labels.

Many retinoid receptors have been found outside of the nucleus and cell body, in various subcellular compartments such as neurites, axons, dendrites and growth cones (Zhelyaznik and Mey, 2006; Chen and Napoli, 2008; Carter et al., 2010, 2011, 2015; Walker et al., 2018a). For example, RARα is translocated from the nucleus to the cytoplasm and exhibits increased axonal expression during hippocampal development (Huang et al., 2008). Extranuclear RAR is also found in neurites and growth cones of cultured embryonic *Xenopus* neurons (Rand et al., 2017) and (together with extranuclear RXRs) in cultured adult neurons from the mollusc, *Lymnaea stagnalis* (Carter et al., 2010, 2015). Even within the adult CNS of *Lymnaea*, the retinoid receptor proteins (RXR/RAR) are found mainly in nerve bundles (Figure 2A) with no detectable expression in the nuclear domains (Carter et al., 2010, 2015), strongly supporting non-canonical functions in this species.

*Xenopus and Lymnaea* growth cones exhibit rapid attractive turning responses to atRA (Dmetrichuk et al., 2006, 2008; Farrar et al., 2009; Carter et al., 2010; Rand et al., 2017; Johnson et al., 2019; Walker et al., 2022), effects that are mimicked by synthetic retinoids and inhibited by retinoid receptor antagonists. Notably, the effects of atRA in *Lymnaea* were not affected by transcriptional inhibitors, indicating a non-genomic mechanism (Farrar et al., 2009). Molluscan neurites survive and grow following physical isolation from their cell body and nucleus (van Kesteren et al., 2006), and atRA and 9-*cis* RA also induced growth cone turning in transected neurites, confirming a non-genomic action and one independent of any influence of the nucleus (Farrar et al., 2009; Carter et al., 2010). These non-genomic chemoattractive effects did however require local protein synthesis and calcium influx (Farrar et al., 2009), as well as the activity of the RhoGTPases, Rac and Cdc42 (Johnson et al., 2019).

#### 5.1.2 Non-canonical role of retinoid actions in the injured CNS

Neurite outgrowth, axonal pathfinding and appropriate target selection are all required for functional regeneration in the adult CNS following injury. Factors that interfere with regenerative capacity in the adult mammalian CNS include the presence of growth inhibitory molecules (He and Koprivica, 2004), lack of growth promoting factors (Schnell et al., 1994; Lu et al., 2004), and/or the intrinsic capacity of injured adult neurons for regeneration (Kwon and Tetzlaff, 2001).

Myelin-associated glycoprotein (MAG) is a transmembrane protein that can inhibit neurite outgrowth in mature neurons (Salzer et al., 1990; DeBellard et al., 1996) and thus inhibit regeneration (Filbin, 1995). Agudo et al. (2010) found that when cultured cerebellar neurons were exposed to both MAG and the RARβ agonist, CD2019, RARβ activation counteracted the inhibitory effects of MAG and enhanced neurite outgrowth. The effects of CD2019 in overcoming the MAG-mediated inhibition, were through a non-canonical pathway mediated by PI3K. Because increased levels of the downstream target of PI3K (phospho-Akt), were also shown in lesioned neurons *in vivo*, the authors proposed that the same non-canonical, PI3K-dependent pathway also likely operates to control axonal outgrowth *in vivo*. Using lesioned rat spinal cords, the authors determined that only injured neurons from CD2019-treated animals showed increased levels of phospho-Akt and exhibited regenerative outgrowth into the lesion site and forelimb innervation site. Despite the non-canonical activation of PI3K/Akt by RARβ, it is unclear whether these *in vivo* effects can be attributed to non-genomic signaling because CD2019 treatment lasted 14 days and transcriptional activation was not directly ruled out.

In the mollusc *Lymnaea*, exogenous retinoids rapidly (15–20 mins) alter the electrical activity of cultured adult neurons (having undergone axotomy injury during removal from the CNS). Such changes were confirmed as non-genomic in nature, as they also occurred in isolated neurites (Vesprini and Spencer, 2014). Interestingly, neurons within the intact CNS exhibited similar rapid changes in firing to retinoids, but only if the CNS had undergone a nerve crush injury 24 h earlier. It is well-known that neuronal firing can affect regenerative responses (Al-Majed et al., 2000) and the authors proposed that the non-genomic, RA-induced changes in firing might induce a favorable intracellular environment to promote regeneration (Vesprini and Spencer, 2014), which may or may not require downstream gene transcription.

#### 5.1.3 The role of microRNAs in RA-mediated regeneration and growth cone guidance

Tail (and spinal cord) amputation in the regeneration-capable adult newt causes dysregulation of various miRNAs, with one example being the downregulation of miR-133a. This downregulation was important for functional regeneration (which miR-133a inhibits by preventing RARβ2 translation). Other predicted targets of miR-133a include CRABP1, RARα and RXRα (Lepp and Carlone, 2014). Despite normal upregulation of RARβ protein following tail amputation, RXRα protein was downregulated, a change also important for regeneration (Walker et al., 2018a). However, as RXRα mRNA levels concomitantly increased, the authors proposed a post-transcriptional, non-canonical regulation of RXRα levels, possibly by miRNAs or other post-transcriptional regulatory mechanisms. They also proposed that miRNAs might reduce cell proliferation and shift cellular fate toward RA-mediated differentiation in regenerating tissues (though this was not tested). Both RARβ and RXRα proteins showed non-overlapping cytoplasmic localization in the regenerating spinal cord and were mostly absent from the cell nuclei. This supports the notion that the receptors might act (possibly independently) in a non-canonical manner during newt spinal cord regeneration (Walker et al., 2018a) (as these receptors normally act as heterodimers to mediate canonical signaling in the nucleus).

MiRNA levels have also been studied following nerve transection in regeneration-capable invertebrate species. Following nerve injury to the *Lymnaea* CNS, miR-124 was upregulated in CNS exposed to RA, which promoted neurite outgrowth, compared to vehicle-treated CNS with no outgrowth. MiR-124 was then shown to be required for RA-induced chemoattraction in cultured neurons (previously shown to be non-genomic) and exhibited varying spatiotemporal changes during the protein synthesis-dependent responses of growth cones to atRA (Walker et al., 2018b, 2022; Figure 2B). There is also evidence for miRNAs controlling local protein synthesis within axons during development (Hancock et al., 2014) and in growth cones responding to other guidance cues (Baudet et al., 2011; Bellon et al., 2017). For example, in cultured embryonic eye explants, pre-miRNAs are trafficked along retinal ganglion axons and locally processed within growth cones in response to the guidance cue semaphorin-3A (Corradi et al., 2020). Whether RA affects the trafficking and processing of pre-miRNAs to mediate local processing of miR-124 during RA-mediated growth cone turning has not yet been determined.

#### 5.1.4 Non-canonical effects of retinoids during intercellular communication

Exosome biogenesis and secretion is a form of intercellular communication regulated by different signaling pathways. Exosomes carry many different molecules from signaling proteins (e.g., growth inhibitory molecules) and lipids, to DNA and RNA, including ncRNAs (Valadi et al., 2007; Guescini et al., 2010; Subra et al., 2010; Andjus et al., 2020). Lin et al. (2021) recently indicated a non-canonical role for atRA-CRABP1 in regulating exosome secretion from cultured neurons, responsible for the transferal of a pro-inflammatory regulator, RIP140 (receptor-interacting protein 140) to macrophages. In the absence of CRABP1 signaling (CRABP1 knockouts), plasma RIP140 increased, suggesting enhanced exosome secretion.

Exosome secretion is normally promoted by growth factor-mediated MAPK signaling. It was proposed by Lin et al. (2021) that in physiological contexts (containing growth factors), RA-CRABP1 dampens this MAPK-mediated exosome secretion. Notably, in the absence of growth factors, exogenous RA enhanced exosome secretion of RIP140 (an effect abolished in CRABP1 knockout cells). This non-canonical RA-CRABP1 signaling pathway was confirmed to be independent of RAR and transcription but did require protein synthesis.

Neuronal exosome release (and subsequent uptake by adjacent glial cells) has also been shown in the context of nerve regeneration and can mediate neurite outgrowth in the injured rat spinal cord (Goncalves et al., 2015, 2018), though a non-genomic role for RA was not examined. However, the RA-CRABP1 mediated non-genomic regulation of exosome secretion (containing the inflammatory signals RIP140; Lin et al., 2021), might have implications for regeneration and might even involve similar mechanisms. For example, inflammatory responses critically affect regenerative capacity in the axolotl; reductions in macrophages and/or alterations in various inflammatory factors result in failed limb regeneration (Lévesque et al., 2007; Godwin et al., 2013).

Above, we discussed a non-canonical role for RARβ in stimulating axon outgrowth through activation of PI3K signaling (Agudo et al., 2010). PI3K/Akt/mTOR signaling is also involved in glial scar formation and is upregulated in reactive astrocytes of the injured spinal cord (Codeluppi et al., 2009; Chen et al., 2016). PTEN (phosphatase tensin homolog, a known inhibitor of PI3K/Akt) is located in the neuronal membrane (Park et al., 2010) where it indirectly reduces neurite outgrowth through PI3K inhibition. Goncalves et al. (2015) show that the RARβ agonist, CD2019, leads to rapid removal of membrane-bound PTEN, resulting in its inactivation and subsequent secretion in exosomes. PTEN is also important in cell proliferation and when PTEN-containing exosomes were injected into an injured rat spinal cord, astrocyte proliferation and glial scarring were reduced. These effects of PTEN-induced PI3K/Akt modulation in the spinal cord were cell-type dependent. Overall, these data suggest that non-canonical retinoid signaling via RARβ, affected both intracellular and intercellular PTEN to promote a permissive environment for axon outgrowth in the injured rat spinal cord.

Goncalves et al. (2018) then showed that neuronal RARβ activation with CD2019, led to increased local RA production in NG2+-expressing glial cells, which was subsequently released in an exosome-associated manner to support a permissive environment for neurite outgrowth. This study did not however determine whether these actions of RARβ were non-genomic in nature, though the signaling that exists between neurons and glial cells might represent a non-canonical function for retinoids and their receptors. Previous studies have shown upregulation of various retinoid receptor proteins (RARα/RXRα) in regenerating rat axons following sciatic nerve crush injury (Zhelyaznik and Mey, 2006), and even though this upregulation may have required transcriptional events, the localization of these receptors in the distal regenerating axons, and their potential ability when activated, to signal to glial cells, might also suggest a non-canonical signaling mechanism.

## 6 Memory and synaptic plasticity: a brief overview of the role of retinoids

It has been known for many decades that retinoic acid and its receptors affect synaptic plasticity (the ability of synaptic connections to change). For example, long-term potentiation (LTP) and long-term depression (LTD), both considered important cellular correlates of learning and memory in the hippocampus, are impaired or eliminated following post-natal Vitamin A deprivation in mice (Misner et al., 2001). Furthermore, knockdown of both RARβ and RARγ subtypes impairs hippocampal plasticity and compromises learning (Chiang et al., 1998). Despite the possibility that many effects of retinoids on long-term memory are mediated by its canonical control of gene transcription, it has become apparent from a number of experimental avenues, that RA is also likely exerting non-canonical and non-genomic effects to mediate changes in synaptic transmission and thus learning and memory.

### 6.1 Non-canonical effects of retinoids on vertebrate synapses

An early study showed retinoid-mediated changes in neurotransmitter release at developing (cultured) neuromuscular junctions of the frog, *Xenopus* (Liao et al., 2004). Dose-dependent enhancement of spontaneous acetylcholine release was observed with atRA (1–30 μM), which was mimicked by RARβ-selective agonists (but not by the precursor of RA, all-*trans* retinol, ruling out a non-specific perturbation of membrane lipids). The rapid onset of effects (5–10 min), likely precluded a genomic mechanism, and later, the authors determined that inhibitors of IP3 and ryanodine receptors, as well as buffering intracellular Ca^2+^ levels, blocked the effects of atRA. Using various kinase inhibitors, the authors proposed that RA likely activated phospholipase C (PLC)/PI3-kinases and Src tyrosine kinase to mediate Ca^2+^ release from IP3 and/or ryanodine-sensitive stores (Liou et al., 2005).

Whereas, Liou et al. (2005) showed that RA increased the frequency of miniature synaptic responses (indicating a pre-synaptic effect on neurotransmitter release), Aoto et al. (2008) showed that atRA (1 μM) rapidly induced changes in the amplitude of miniature synaptic currents (within 1 h), indicating direct effects on the post-synaptic neuron. Because no evidence for new dendritic spine formation was found, these data suggested that atRA acted on existing synapses. Though primary cultures of dissociated hippocampal neurons were used, these are a well-established model for examining synaptic plasticity (LTP and LTD; Fitzsimonds et al., 1997; Bi and Poo, 1998; Tao et al., 2000), and the retinoid-mediated effects were then also confirmed in brain slices.

The authors next determined that these effects of RA were important for homeostatic plasticity, a mechanism by which neurons restore synaptic activity following its reduction (Aoto et al., 2008). Such up-scaling of synaptic activity was prevented when RA synthesis was inhibited. The authors subsequently demonstrated (using both cell culture and hippocampal slices), that blockade of neuronal activity enhanced RA synthesis, which led to an increase in GluR1 (glutamate receptor subunit) expression in the post-synaptic membrane, which mediated the amplitude increases in the post-synaptic responses. Due to the rapid effects of RA (unaffected by a transcriptional inhibitor), as well its ability to mediate these effects in preparations lacking nuclei (synaptoneurosomes), the authors convincingly demonstrated non-genomic effects of RA.

It is noteworthy that the neuronal activity-blockade (that induced downstream RA synthesis and enhanced GluR1 expression), was able to occlude any effects of exogenously applied 1 μM RA, indicating that this concentration of exogenous RA was not capable of inducing effects beyond those produced physiologically (Aoto et al., 2008). These authors also provided evidence that under basal conditions, levels of RA might be very low, suggesting that attempts to measure endogenous levels of RA in the adult brain might not actually represent levels of RA that might be produced “on demand” in synaptic microdomains (such as pre-synaptic axon terminals or post-synaptic dendrites). As highlighted above, the RA levels that are generated “on demand” during synaptic changes, appear to be sufficient to occlude the subsequent effects of exogenously applied 1 μM RA.

The same research group showed that RARα can be actively exported from the nucleus and is found in dendritic domains, where it acts as an RNA binding protein (Poon and Chen, 2008). The authors again used synaptoneurosomes, in addition to whole hippocampal tissue, to examine the specific nature of RARα binding to various mRNA species. In the absence of RA (and presumably under very low, basal levels of RA), RARα is thought to repress translation of GluR1 by binding to its UTR (untranslated region of the mRNA), a repression that is subsequently relieved by exogenous RA (Poon and Chen, 2008). Khatib et al. (2019b) also confirmed that RA induces GluR1 translation, using a reporter assay under the control of the GluR1 5′ UTR bound by RAR in SH-SY5Y cells. A number of synthetic RXR and RAR ligands were also screened, only a subset of which were found to activate GluR1. This reporter for RAR-dependent translational control of the GluR1 might provide a useful tool for generation of therapeutic RARα-selective ligands that specifically target synaptic plasticity (Khatib et al., 2019b).

Other studies by the Chen group linked dendritic RA synthesis to calcium levels (Wang et al., 2011). Under basal post-synaptic levels of calcium, RA synthesis is repressed, but when reduced neuronal activity leads to reduced calcium entry (through either glutamate receptors or L-type Ca channels), RA synthesis is induced (though how, is not clear). Dendritic localization of RARα in mouse hippocampal neurons was also independently verified by Chen and Napoli (2008), and GluR1 expression was again upregulated by RA. These authors provided additional evidence that within their experimental parameters, dendritic growth also occurred within 30 min of applied atRA. They further showed that concentrations of atRA as low as 10 nM could increase ERK1/2 phosphorylation within 10 min, importantly demonstrating that non-canonical effects of RA can also occur at low, nanomolar concentrations.

In addition to the ability of RA to scale up synaptic excitation, Sarti et al. (2013) also demonstrated an independent (but parallel) role of RA in rapid *down*-scaling of synaptic *inhibition*. Surface biotinylation experiments indicated that both RA and neuronal activity-blockade reduced surface expression of GABA_A_ receptors (a class of chloride ion channels that mediate fast synaptic inhibitory responses to GABA). Once again, this effect was dependent on RA synthesis and independent of gene transcription (but not translation), indicating a non-genomic function. Interestingly, recordings revealed that RA can boost the excitatory/inhibitory balance when neurons are in a depolarized state, and by independently regulating both excitatory (glutamatergic) and inhibitory (GABAergic) synapses, the authors proposed that RA might act as a “master organizer” of neuronal activity, at least in the hippocampus. Of note, is that synapses in other brain areas are similarly affected by RA; atRA increases spontaneous excitatory synaptic currents in a transcriptionally-independent manner in mouse cortex, as well as in human cortical slices, indicating for the first time that RA-induced effects on synaptic plasticity may also occur in the human brain (Lenz et al., 2021).

In addition to RA inducing rapid effects in the post-synaptic neuron, it might indirectly affect neurotransmitter release from the pre-synaptic cell (Thapliyal et al., 2022). Synaptoneurosomes treated with 1 μM atRA (for 30 min) not only increased GluR1 levels, but also increased expression of proBDNF (Figure 1B). It was determined that RARα exhibited specific binding to two dendritically localized *Bdnf* transcripts, thus expanding the scope of mRNA binding and translational control by dendritically localized RARα. It was proposed that enhanced RA synthesis increased translation of BDNF, which then acted retrogradely on pre-synaptic terminals (via TrKB receptors) to increase neurotransmitter release.

Although many studies have suggested that memory impairments result from reductions in RA signaling, a recent study by Wołoszynowska-Fraser et al. (2021) provided paradoxical evidence that increases in hippocampal RA signaling were associated with memory impairments in aged rats. They found increased levels of RARα in hippocampal synaptosomes, implying non-genomic actions of RA were involved. The mechanisms linking changes in retinoid signaling with the age-related memory deficits are not yet known, though a possible disruption in the excitatory/inhibitory balance was proposed. These studies also emphasize the importance of future considerations of age-related changes in non-genomic RA signaling.

### 6.2 Non-canonical effects of retinoids in invertebrate neurons

Though retinoids are well-known to affect synaptic plasticity and learning and memory in many vertebrates (such as songbirds and rodents; Chiang et al., 1998; Denisenko-Nehrbass et al., 2000; Wood et al., 2008), studies in the mollusc *Lymnaea stagnalis* have indicated a conserved role of retinoids in memory formation in invertebrates (Rothwell and Spencer, 2014; Rothwell et al., 2014; Carpenter et al., 2016). Studies indicated that inhibitors of RA synthesis and retinoid receptor antagonists, prevented long-term (24 h) memory formation (but not learning or intermediate memory), whereas receptor agonists promoted the formation of long-term memory and enhanced its duration. These findings indicate that retinoids can influence memory formation in invertebrates as well as vertebrates.

An examination of cellular effects in molluscan neurons (*Lymnaea stagnalis*) indicated that RA induced changes in electrical properties as well as calcium signaling. One and ten μM atRA induced rapid spike broadening and/or plateau potentials (prolonged periods of stable depolarization), and neurons transitioned from tonic to burst firing, often falling silent within 35 min (Vesprini and Spencer, 2014). This effect was specific to atRA and was not induced by the precursor retinol, or by the isomer, 9-*cis* RA. Even though the 9-*cis* RA isomer has proven difficult to detect in vertebrates, it is present in similar concentrations to atRA in the *Lymnaea* CNS (Dmetrichuk et al., 2008). The rapid effects of atRA on cell firing were reduced (but not abolished) by an RXR-selective antagonist, HX531, suggesting an involvement of non-nuclear retinoid receptors (RXR and RAR are only detected in non-nuclear domains of adult molluscan neurons, both in the CNS and in culture; Carter et al., 2010, 2015). These rapid effects of atRA on firing activity were, however, unaffected by inhibitors of protein synthesis, protein kinase A (PKA) or PLC (Vesprini et al., 2015).

Importantly, it was determined that atRA also induced rapid, atypical firing in transected neurites (in the physical absence of the nucleus), which ruled out any genomic effects (in a similar manner as the use of synaptoneurosomes in vertebrate neurons). To rule out a cell culture artifact (a fair criticism raised concerning studies of non-genomic effects of retinoids), atRA was also shown to induce changes in action potential shape in the same neurons within the intact CNS (Vesprini and Spencer, 2014). The atRA-induced changes in firing properties were later shown to be activity-dependent, with the underlying mechanism involving enhanced inactivation of delayed rectifier voltage-gated K^+^ channels (de Hoog and Spencer, 2022), though this latter study used longer exposure times to atRA, not yet ruling out a genomic component to RA-mediated regulation of K^+^ channels.

In the same neurons, atRA was also shown to rapidly reduce intracellular calcium levels (within 15 min), in a similar dose- and isomer-dependent manner as the changes in firing activity (Vesprini et al., 2015). However, unlike changes in firing activity, changes in calcium levels were not affected by the receptor antagonist HX531, suggesting a receptor-independent mechanism. atRA was then shown to rapidly reduce ion flux through voltage-gated calcium channels (VGCCs); within 20 min, atRA significantly reduced the peak current and increased the inactivation rate of VGCCs, meaning the channels were now passing less current and for shorter periods of time. Though changes in current flow occurred over a similar time course as the changes in firing activity, they were deemed unlikely the cause, suggesting that RA might exert multiple, divergent non-canonical effects within a neuron, and over a similar time course.

Although nanomolar concentrations of atRA are often sufficient to elicit activation of nuclear retinoid receptors (canonical effects), many non-canonical effects require micromolar concentrations of RA. In the molluscan CNS, RA levels are estimated to be as high as 0.6 μM (Dmetrichuk et al., 2008), suggesting that, at least for molluscan (*Lymnaea*) neurons, low micromolar RA concentrations might be within a “physiological” range. Despite longer RA exposure times, de Hoog et al. (2018) later determined that atRA concentrations as low as 0.5 μM were capable of reducing the peak current through VGCCs.

de Hoog et al. (2019) showed that acute application of retinoid receptor antagonists also inhibited ion flux through VGCCs, suggesting retinoid receptors might be constitutively active in *Lymnaea* neurons. Specifically, RXR (HX531) and RAR (LE540) antagonists exerted rapid voltage-dependent inhibition of ICa mediated by Ca_v_2s (channel subtypes generally responsible for neurotransmitter release). Voltage-dependent inhibition of VGCCs is a phenomenon found in vertebrate neurons, important for synaptic plasticity, and resulting from G-protein binding to VGCCs. The voltage-dependent inhibition mediated by LE540 in molluscan neurons was also G-protein dependent, providing additional evidence for retinoid receptor interactions with G-proteins. Interestingly, the voltage-dependent inhibition produced by HX531 was independent of G-proteins and might thus indicate the involvement of a novel mechanism.

Together these studies indicated that retinoid signaling can rapidly and non-canonically modulate cell firing and fine-tune Ca^2+^ signaling by Ca_v_2 channels, though the outcome on synaptic function or plasticity in the molluscan CNS has not yet been determined. It is also not yet known whether RA or its receptors can modulate neuronal voltage-gated Ca^2+^ or K^+^ channels in vertebrate neurons.

## 7 Perspectives

Here, we have discussed evidence for non-canonical effects of RA, which include direct interactions with signaling cascades or actions via non-nuclear retinoid receptors. These non-canonical actions appear to be important for many neuronal processes, including development, regeneration and synaptic plasticity (underlying learning and memory). However, it should be noted that these physiological processes can also require transcriptional control by retinoids, and the extent to which canonical (transcriptional) and non-canonical (non-transcriptional) actions of retinoids potentially interact, needs further study. There are indications that some processes, such as neurite outgrowth (Khatib et al., 2019a) might require both, whereas others, such as growth cone guidance (Farrar et al., 2009), might require only localized effects of RA. There are many other questions that will need to be addressed in the future regarding non-canonical retinoid signaling, and these include how retinoid signaling might be induced in local domains, such as axons and dendrites. There is currently evidence that neuronal activity and/or calcium levels might regulate RA synthesis in hippocampal dendrites (Aoto et al., 2008; Wang et al., 2011), but studies in other cell types and physiological contexts will be needed. Furthermore, whether the extra-nuclear receptors differ from those in the nucleus also requires closer examination, as well as how the synthesis or transport of extra-nuclear receptors to axons or dendrites might be mediated and/or controlled.

The current paucity of direct evidence for non-genomic activity by *endogenous* retinoids, have led some to suggest that non-canonical or non-genomic retinoid signaling might not occur *in vivo* (Duester, 2022, 2023). Duester suggests that alternative strategies to investigate non-genomic signaling, particularly during synaptic plasticity, should include removal of endogenous RA using genetic knockouts of RA-synthesizing enzymes. Although such approaches might validate the *requirement* for RA in, for example, homeostatic plasticity, they would not likely determine whether such roles are mediated solely by non-genomic mechanisms. Vitamin A-deficient diets (Misner et al., 2001) and retinoid receptor knockouts (Chiang et al., 1998) have previously confirmed a role for RA in LTP and LTD (also forms of synaptic plasticity), though did not distinguish between genomic and possible non-genomic actions. Cell-type specific (conditional) knockouts might however provide information on the origin (or site of action) of RA in the hippocampus or other regions of the adult brain, during the examination of its physiological effects.

It should be noted that there are inherent difficulties in studying potential non-genomic mechanisms *in vivo*. For example, synaptic sites are more difficult to access, growth cones difficult to image, and exosomes more difficult to obtain. Though the use of cell culture and *in vitro* experiments might not reliably mimic *in vivo* conditions, many experiments studying LTP, LTD and homeostatic plasticity in cultured neurons, can be reliably reproduced in the hippocampus. The same applies to the role of various guidance cues that have been examined both *in vitro* and *in vivo*. As such, despite the requirement to eventually reproduce findings *in vivo, in vitro* assays might still provide promising insights into potential non-genomic effects of retinoids.

The concentration of RA required to exert non-genomic effects can vary and may be higher than those that exert genomic effects (Khatib et al., 2019a). Indeed, most non-canonical studies have utilized micromolar retinoid concentrations, which have been described as “pharmacologic” (Duester, 2023). However, it is difficult to determine what the physiological levels of RA might be, especially using techniques that generally require large amounts of tissue, such as HPLC/MS. This is especially true for examining RA levels in microdomains such as axon terminals and dendritic spines, where local synthesis of RA might raise the concentrations much faster and to far higher levels than in larger cellular compartments such as cell bodies. Indeed, the effects of exogenous 1 μM atRA on synaptic responses were obscured by physiological induction of RA synthesis (Aoto et al., 2008), supporting the possibility that micromolar concentrations might naturally be reached in neuronal microdomains.

The presence of retinoid receptors within non-nuclear microdomains of the nervous system and the recent identification of a unique cytoplasmic receptor isoform lacking a DNA-binding domain (albeit in T cells) (Larange et al., 2023), supports a non-genomic role for retinoid receptors outside of the nucleus, but also raises questions as to whether receptors in non-nuclear domains are structurally and functionally different from nuclear receptors. The use of preparations that lack nuclei, such as vertebrate synaptoneurosomes, or invertebrate isolated neurites, have provided evidence that extranuclear retinoid receptors remain functional, though these preparations have currently only been examined *in vitro*. Invertebrate neurites can however, be isolated from their cell bodies, even in the intact animal and CNS (Haque et al., 2006) providing the potential for future examination of non-genomic RA signaling *in vivo*.

It is not yet known whether retinoid receptor proteins are trafficked to neuronal microdomains from the nucleus or are synthesized locally, though this might be cell or context-dependent. Alternative and more direct methods for testing the translation of endogenous proteins or reporters at the single mRNA level (such as SunTag or SUnSET), could be used to further investigate the regulation of retinoid receptor synthesis (Blake et al., 2024).

RA can also alter the expression of various post-transcriptional regulators, such as miRNAs, though whether this involves transcriptional changes has not always been clear. As such, the use of fluorescent sensors that can report real time processing of pre-miRNAs during RA-mediated processes will prove useful. For example, a fluorescent sensor, such as that utilized by Sambandan et al. (2017), where a fluorophore reporter attached to the miRNA backbone is released following cleavage of the pre-miRNA, could provide insights into how miRNAs are regulated by RA.

Non-canonical retinoid signaling is not limited to cell-autonomous processes, as it also plays a role in exosome secretion. Understanding how retinoids might control intercellular communication can be further examined using non-targeted, top-down approaches, such as RNA sequencing and proteomics, rather than targeted approaches which only examine candidate molecules (Carbonara et al., 2021). Exosomes are secreted following neuronal injury, and of note, is that exosome content can differ between animals with variable regeneration competency (i.e., urodele vs. mammals) (Middleton et al., 2018). Studying mTOR, an identified target of non-genomic retinoid signaling, might also further our understanding of species-specific regenerative competency, as unique mTOR variants exist within urodele amphibians, and might be responsible for the rapid translation of pre-existing mRNAs required for regeneration following injury (Zhulyn et al., 2023).

Further examination of non-canonical *endogenous* retinoid signaling across species will allow us to gain a better understanding of the evolution and diversity of retinoid signaling, as well as lead to the potential identification of novel and specific targets for drug design, which may include CRABP1. For example, there are reports of disease-associated overactivation of ERK (cancer; Persaud et al., 2016) and CaMKII (i.e., cytotoxicity induced cardiomyopathy and motor neuron degeneration/loss) (Park et al., 2018; Lin et al., 2022), both of which are downstream targets of CRABP1 and non-canonical RA signaling. In order to specifically target CRABP1, selective ligands have been designed for binding pockets on CRABP1 (which have little structural or sequence similarity with those of RAR), though future studies need to rule out potential off-target effects (Newcomer, 1993; Newcomer et al., 1993; Kleywegt et al., 1994; Nhieu et al., 2023). Interestingly, such CRABP1-selective ligands have been shown to mitigate motor neuron degeneration (Nhieu et al., 2023). In order to therapeutically target only non-canonical RA signaling (and avoid canonical transcriptional effects), we thus need a better understanding of such non-canonical mechanisms and the roles they might play in nervous system injury and/or disease (Clark et al., 2020; García-Padilla et al., 2021; Lavudi et al., 2023; Nhieu et al., 2023).

## Author contributions

AP: Conceptualization, Writing—original draft, Writing—review & editing. RC: Conceptualization, Funding acquisition, Writing—original draft, Writing—review & editing. GS: Conceptualization, Funding acquisition, Writing—original draft, Writing—review & editing.
